# Modulo Periodic Poisson Stable Solutions of Quasilinear Differential Equations

**DOI:** 10.3390/e23111535

**Published:** 2021-11-18

**Authors:** Marat Akhmet, Madina Tleubergenova, Akylbek Zhamanshin

**Affiliations:** 1 Department of Mathematics, Middle East Technical University, Ankara 06800, Turkey; akylbek78@mail.ru; 2 Department of Mathematics, Aktobe Regional University, Aktobe 030000, Kazakhstan; madina_1970@mail.ru

**Keywords:** modulo periodic Poisson stable functions, quasilinear differential equations, modulo periodic Poisson stable solutions, asymptotic stability

## Abstract

In this paper, modulo periodic Poisson stable functions have been newly introduced. Quasilinear differential equations with modulo periodic Poisson stable coefficients are under investigation. The existence and uniqueness of asymptotically stable modulo periodic Poisson stable solutions have been proved. Numerical simulations, which illustrate the theoretical results are provided.

## 1. Introduction

The theory of differential equations is a doctrine on oscillations and recurrence, which are basic in science and technique. Oscillations are most preferable in engineering [1], while recurrence originates in celestial mechanics [2]. The ultimate recurrence is the Poisson stability [3,4,5]. Presently, needs for functions with irregular behavior are exceptionally strong in neuroscience and celestial dynamics, which is still in the developing mode. In the present research, we have decided to combine periodic dynamics with the phenomenon of Poisson stability. That is, one of simplest forms of oscillations is amalgamated with the most sophisticated type of recurrence. We hope that the choice can give a new push for the nonlinear analysis, which faces challenging problems of the real world and industry. The present product of the design are *modulo periodic Poisson stable functions*.

In paper [6], to strengthen the role of recurrence as a chaotic ingredient we have extended the Poisson stability to the unpredictability property. Thus, the Poincaré chaos has been determined, and one can say that the *unpredictability implies chaos* now. The unpredictable point of the Bebutov dynamics is the unpredictable function. In papers [7,8,9,10,11,12,13,14,15], we provided a dynamical method, how to construct Poisson stable functions. Deterministic and stochastic dynamics have been used. Deterministically unpredictable functions have been constructed as solutions of hybrid systems, consisting of discrete and differential equations [9,13,14], and randomly they are results of the Bernoulli process inserted into a linear differential equation [7,10,16]. Unpredictable oscillations in neural networks have been researched in [7,13,17,18,19].

In papers [8,9,10,14] and books [7,13], discussing existence of unpredictable solutions, we have developed a new method how to approve Poisson stable solutions, since unpredictable functions are a subset of Poisson stable functions, and to verify the unpredictability one must check, if the Poisson stability is valid. The method is distinctly different than the *comparability method by character of recurrence*, which was introduced in [20] and later has been realized in several articles [21,22,23,24,25,26,27]. Unlike papers [7,8,9,10,13,14,15,16,17,18,19], the present research is busy with the new type of Poisson stable functions. Correspondingly, it is the first time in literature, when quasilinear equations with Poisson stable coefficients are under investigation. Finally, the systems are approved with modulo periodic Poisson stable solutions. The newly invented method of verification of the Poisson stability joined with the presence of the periodic components in the recurrence has made possible the extension for the class of studied differential equations. In papers [21,22,23,24], quasilinear systems are with constant matrices of coefficients, and in our case, we research systems with periodic and, even with Poisson stable coefficients. Another significant novelty is the numerical simulation of the Poisson stable functions and solutions [7,9,13,14]. We believe that altogether, the present suggestions can shape a new interesting science direction, not only in the theoretical study of differential equations, but also they provide rich opportunities for applications in mechanics, electronics, artificial neural networks, neuroscience.

## 2. Preliminaries

Throughout the paper, R and N will stand for the set of real and natural numbers, respectively. Additionally, the norm ${∥u∥}_{1}=\sup_{t\in R}∥u\left(t\right)∥,$ where $∥u∥=\max_{1\leq i\leq n}\left|u_{i}\right|,$ $u=\left(u_{1},\ldots ,u_{n}\right),u_{i}\in R,i=1,2,...,n,$ will be used. Correspondingly, for a square matrix $A=\{a_{ij}\},i,j=1,2,...,n,$ the norm $∥A∥=\max_{i=1,\ldots ,n}\sum_{j=1}^{n}\left|a_{ij}\right|$ will be used.

**Definition** **1** ([5])**.** *A continuous and bounded function $\psi \left(t\right):R\to R^{n}$ is called Poisson stable, if there exists a sequence $t_{k},$ which diverges to infinity such that the sequence $\psi (t+t_{k})$ converges to ψ(t) uniformly on bounded intervals of R.*

The sequence $t_{k}$ in the last definition is said to be *Poisson sequence* of the function ψ(t).

By Lemma A1 in the Appendix A, for a positive fixed ω there exist a subsequence $t_{k_{l}}$ of the Poisson sequence $t_{k}$ and a number $\tau_{\omega }$ such that $t_{k_{l}}\to \tau_{\omega }\left(mod\;\omega \right)$ as l→∞. We shall call the number $\tau_{\omega }$ as the *Poisson shift* for the Poisson sequence $t_{k}$ with respect to the ω. It is not difficult to find that for the fixed ω the set of all Poisson shifts, $T_{\omega },$ is not empty, and it can consist of several and even infinite number elements. The number $\kappa_{\omega }=inf\;T_{\omega },$ $0\leq \kappa_{\omega }<\omega ,$ is said to be *the Poisson number* for the Poisson sequence $t_{k}$ with respect to the number ω.

**Definition** **2.** *The sum ϕ(t)+ψ(t) is said to be a modulo periodic Poisson stable (MPPS) function, if ϕ(t) is a continuous periodic and ψ(t) is a Poisson stable functions.*

We shall call the function ϕ(t) the *periodic component* and the function ψ(t) the *Poisson component* of the MPPS function in what follows.

**Remark** **1.** *Duo to Lemma A3, an MPPS function is a Poisson stable if $\kappa_{\omega }$ equals zero. Otherwise, without loss of generality, the sequence $\phi \left(t+t_{k}\right)+\psi \left(t+t_{k}\right)$ converges on all compact subsets of the real axis to the function $\phi \left(t+\tau_{\omega }\right)+\psi \left(t\right),$ where $\tau_{\omega }$ is a nonzero Poisson shift for the sequence $t_{k}.$ Since of the periodicity of the function ϕ(t), one can accept the last convergence as a special form of recurrence. In the next section, we shall consider it as a result of Theorem 1.*

## 3. Main Results

### 3.1. Linear System of Differential Equations

Consider the following system

(1) $$\begin{array}{c} x^{\prime }\left(t\right)=A\left(t\right)x\left(t\right)+\phi \left(t\right)+\psi \left(t\right), \end{array}$$

where t∈R, $x\in R^{n},$ n∈N, $\phi \left(t\right):R\to R^{n}$ and $\psi \left(t\right):R\to R^{n}$ are continuous functions, A(t) is a continuous n×n matrix.

We assume that the following conditions are satisfied.

**(C1)** 
A(t) is an ω− periodic matrix for a fixed positive ω;
**(C2)** 
ϕ(t) is an ω− periodic function, and ψ(t) is a Poisson stable function with a Poisson sequence $t_{k};$
**(C3)** 
the Poisson number $\kappa_{\omega }$ for the sequence $t_{k}$ is equal to zero.

According to Definition 2 and condition (C2), the sum ϕ(t)+ψ(t) is an MPPS function, i.e., the linear system (1) is with MPPS perturbation.

Let us consider the homogeneous system, associated with (1),

(2) $$\begin{array}{c} x^{\prime }\left(t\right)=A\left(t\right)x\left(t\right). \end{array}$$

Let X(t), t∈R, is the fundamental matrix of the system (2) such that X(0)=I, and *I* is the n×n identical matrix. Moreover, X(t,s) is transition matrix of the system (2), which equal to $X\left(t\right)X^{-1}\left(s\right),$ and X(t+ω,s+ω)=X(t,s) for all t,s∈R.

We assume that the following additional assumption is valid.

**(C4)** 
The multipliers of the system (2) in modulus are less than one.

It follows from the last condition that there exist positive numbers K≥1 and α such that

(3) $$\begin{array}{c} ∥X\left(t,s\right)∥\leq Ke^{-\alpha (t-s)}, \end{array}$$

for t≥s [28].

**Lemma** **1.** *If the inequality (3) is satisfied, then the following estimation is correct*

(4) $$\begin{array}{ccc} & & ∥X\left(t+\tau ,s+\tau \right)-X\left(t,s\right)∥\leq \max_{t\in R}∥A\left(t+\tau \right)-A\left(t\right)∥\frac{2K^{2}}{\alpha^{2}e}e^{-\frac{\alpha }{2}\left(t-s\right)}, \end{array}$$

*for t≥s and arbitrary real number τ.*

**Proof.**  Since

$$\begin{array}{ccc} & & \frac{dX(t+\tau ,s+\tau )}{dt}=A\left(t\right)X\left(t+\tau ,s+\tau \right)+\left(A\left(t+\tau \right)-A\left(t\right)\right)X\left(t+\tau ,s+\tau \right), \end{array}$$

we have that

$$\begin{array}{ccc} & & X\left(t+\tau ,s+\tau \right)=X\left(t,s\right)+\int_{s}^{t}X\left(t,u\right)\left(A\left(u+\tau \right)-A\left(u\right)\right)X\left(u+\tau ,s+\tau \right)du. \end{array}$$

That is why,

$$\begin{array}{ccc} & & ∥X(t+\tau ,s+\tau )-X(t,s)∥\leq \\ & & \int_{s}^{t}∥X\left(t,u\right)∥∥A\left(u+\tau \right)-A\left(u\right)∥∥X\left(u+\tau ,s+\tau \right)∥du\leq \\ & & \max_{t\in R}∥A\left(t+\tau \right)-A\left(t\right)∥\int_{s}^{t}K^{2}e^{-\alpha (t-s)}du=\\ & & \max_{t\in R}∥A\left(t+\tau \right)-A\left(t\right)∥\frac{K^{2}}{\alpha }e^{-\alpha (t-s)}\left(t-s\right)=\\ & & \max_{t\in R}∥A\left(t+\tau \right)-A\left(t\right)∥\frac{K^{2}}{\alpha }e^{-\frac{\alpha }{2}\left(t-s\right)}e^{-\frac{\alpha }{2}\left(t-s\right)}\left(t-s\right). \end{array}$$

Since $\underset{u\geq 0}{\sup}e^{-\frac{\alpha }{2}u}u=\frac{2}{\alpha e},$ the lemma is proved.  □

**Theorem** **1.** *Assume that conditions (C1), (C2) and (C4) are valid. Then the system (1) admits a unique asymptotically stable MPPS solution.*

**Proof.**  The bounded solution of system (1) has the form [28]

(5) $$\begin{array}{ccc} & & x\left(t\right)=\int_{-\infty }^{t}X\left(t,s\right)\left[\phi \left(s\right)+\psi \left(s\right)\right]ds,\;t\in R. \end{array}$$

One can write that $x\left(t\right)=x_{\phi }\left(t\right)+x_{\psi }\left(t\right),$ where $x_{\phi }\left(t\right)=\int_{-\infty }^{t}X\left(t,s\right)\phi \left(s\right)ds$ and $x_{\psi }\left(t\right)=\int_{-\infty }^{t}X\left(t,s\right)\psi \left(s\right)ds.$

It is not difficult to show that the function $x_{\phi }\left(t\right)$ is ω− periodic [29].

Next, we prove that the function $x_{\psi }\left(t\right)$ is Poisson stable. Fix arbitrary positive number ϵ and interval [a,b], −∞<a<b<∞. We will show that for a large *k* it is true that $∥x_{\psi }\left(t+t_{k}\right)-x_{\psi }\left(t\right)∥<\epsilon$ on [a,b]. Let us choose two numbers *c* and ξ such that c<a and ξ is positive, satisfying the following inequalities,

(6) $$\begin{array}{ccc} & & \frac{4K^{2}m_{\psi }}{\alpha^{3}e}\xi <\frac{\epsilon }{3}, \end{array}$$

(7) $$\begin{array}{ccc} & & \frac{2Km_{\psi }}{\alpha }e^{-\alpha (a-c)}<\frac{\epsilon }{3}, \end{array}$$

and

(8) $$\begin{array}{ccc} & & \frac{K\xi }{\alpha }\left[1-e^{-\alpha (b-c)}\right]<\frac{\epsilon }{3}, \end{array}$$

with $m_{\psi }=\underset{t\in R}{\sup}∥\psi \left(t\right)∥.$ By applying condition (C4), without loss of generality, for sufficiently large *k* we obtain that $∥A\left(t+t_{k}\right)-A\left(t\right)∥<\xi$ for all t∈R, and $∥\psi \left(t+t_{k}\right)-\psi \left(t\right)∥<\xi$ for t∈[c,b]. Using Lemma 1 we attain that

$$\begin{array}{ccc} & & ∥x_{\psi }\left(t+t_{k}\right)-x_{\psi }\left(t\right)∥\;=\;∥\int_{-\infty }^{t}\left(X\left(t+t_{k},s+t_{k}\right)\psi \left(s+t_{k}\right)-X\left(t,s\right)\psi \left(s\right)\right)ds∥\leq \\ & & \int_{-\infty }^{t}∥X\left(t+t_{k},s+t_{k}\right)-X\left(t,s\right)∥∥\psi \left(s+t_{k}\right)∥ds\;+\\ & & \int_{-\infty }^{t}∥X\left(t,s\right)∥∥\psi \left(s+t_{k}\right)-\psi \left(s\right)∥ds=\\ & & \int_{-\infty }^{t}∥X\left(t+t_{k},s+t_{k}\right)-X\left(t,s\right)∥∥\psi \left(s+t_{k}\right)∥ds\;+\\ & & \int_{-\infty }^{c}∥X\left(t,s\right)∥∥\psi \left(s+t_{k}\right)-\psi \left(s\right)∥ds\;+\int_{c}^{t}∥X\left(t,s\right)∥∥\psi \left(s+t_{k}\right)-\psi \left(s\right)∥ds\leq \\ & & \int_{-\infty }^{t}\frac{2K^{2}\xi }{\alpha^{2}e}e^{-\frac{\alpha }{2}\left(t-s\right)}m_{\psi }ds+\int_{-\infty }^{t}2Ke^{-\alpha (t-s)}m_{\psi }ds+\int_{-\infty }^{t}Ke^{-\alpha (t-s)}\xi ds\leq \\ & & \frac{4K^{2}\xi }{\alpha^{3}e}m_{\psi }+\frac{2Km_{\psi }}{\alpha }e^{-\alpha (a-c)}+\frac{K\xi }{\alpha }\left[1-e^{-\alpha (b-c)}\right]. \end{array}$$

Now, the inequalities (6) to (8) imply that $∥x_{\psi }\left(t+t_{k}\right)-x_{\psi }\left(t\right)∥<\epsilon ,$ for t∈[a,b]. Therefore, the sequence $x_{\psi }\left(t+t_{k}\right)$ uniformly converges to $x_{\psi }\left(t\right)$ on each bounded interval. Thus, according to the Definition 2 the solution x(t) of the system (1) is MPPS function with the periodic component $x_{\phi }\left(t\right)$ and the Poisson component $x_{\psi }\left(t\right).$ The asymptotic stability of the MPPS solution can be verified in the same way as for the bounded solution of a linear inhomogeneous system [29].  □

The following examples show the validity of the obtained theoretical result.

**Example** **1.** *Let us consider the following linear inhomogeneous system,*

(9) $$\begin{array}{c} \begin{array}{c} x_{1}^{\prime }=\left(-1+0.5sin\left(2t\right)\right)x_{1}+2.5cos\left(t\right)+5.5\Theta^{2}\left(t\right),\\ x_{2}^{\prime }=\left(-2+0.25cos\left(t\right)\right)x_{2}+2sin\left(2t\right)+1.7\Theta \left(t\right), \end{array} \end{array}$$

**Figure 1:**
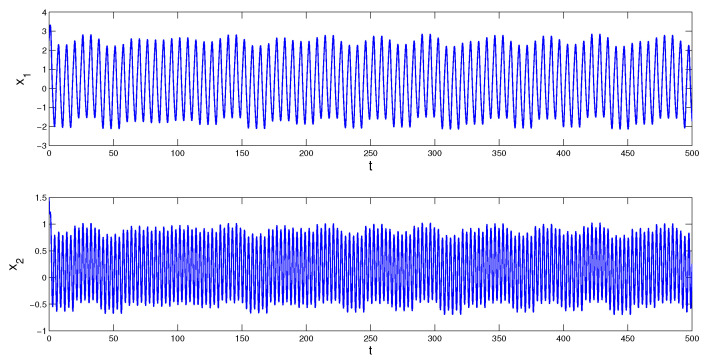
Coordinates of the solution x(t) of system (9) with initial values $x_{1}\left(0\right)=2.5$ and $x_{2}\left(0\right)=1.5,$ which asymptotically converge to the coordinates of the MPPS solution z(t) of the system.

**Figure 2:**
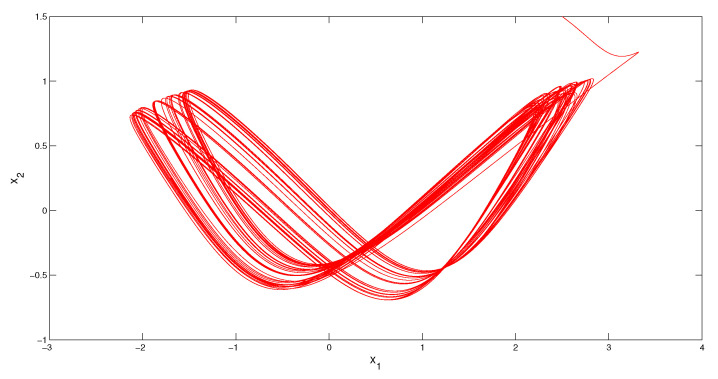
The trajectory of the solution x(t) of the Equation (9), which asymptotically approaches the MPPS solution z(t) of the system.

*where $\Theta \left(t\right)=\int_{-\infty }^{t}e^{-3(t-s)}\Omega_{\left(3.85;6\pi \right)}\left(s\right)ds$ is the Poisson stable function described in Appendix B. The perturbation is an MPPS function with the periodic component $\phi \left(t\right)={\left(2.5cos(t),2sin(2t)\right)}^{T}$ and the Poisson component $\psi \left(t\right)={\left(5.5\Theta^{2}\left(t\right),1.7\Theta \left(t\right)\right)}^{T}.$ The common period of the coefficient A(t) and the periodic component ϕ(t) is 2π. Since the function $\Omega_{\left(3.85,6\pi \right)}\left(t\right)$ is constructed on the intervals [6πi,6π(i+1)), i∈Z, for the Poisson sequence $t_{k}$ of the function Θ(t) there exists a subsequence $t_{k_{l}}$ such that $t_{k_{l}}\to 0\left(mod\;2\pi \right).$ Therefore, the Poisson number $\kappa_{\omega }=0.$ Condition (C4) is valid with the multipliers $\rho_{1}=e^{-2\pi },$ and $\rho_{2}=e^{-4\pi }.$ According to Theorem 1, the system admits a unique asymptotically stable MPPS solution, z(t). Since it is impossible to determine the initial value of the solution, we simulate a solution, which asymptotically approaches z(t) as time increases. We depict in Figure 1 the coordinates of the solution x(t), with initial values $x_{1}\left(0\right)=2.5$ and $x_{2}\left(0\right)=1.5,$ which visualizes the MPPS solution approximately. In Figure 2 the trajectory of the solution x(t) is shown.*

**Figure 3:**
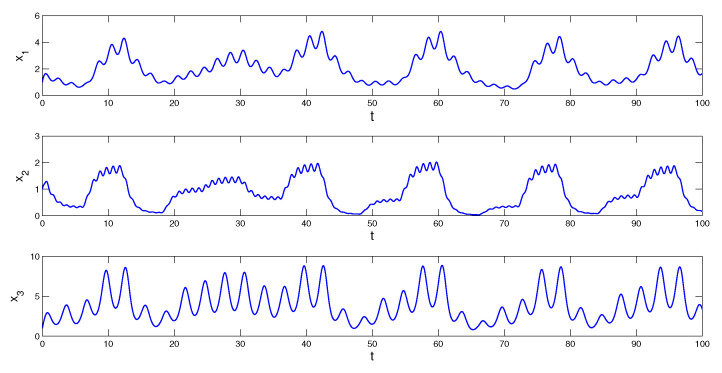
Coordinates of the solution x(t), with initial values $x_{1}\left(0\right)=1,$ $x_{2}\left(0\right)=1$ and $x_{3}\left(0\right)=1,$ which asymptotically converge to the coordinates of the MPPS solution of system (10).

**Figure 4:**
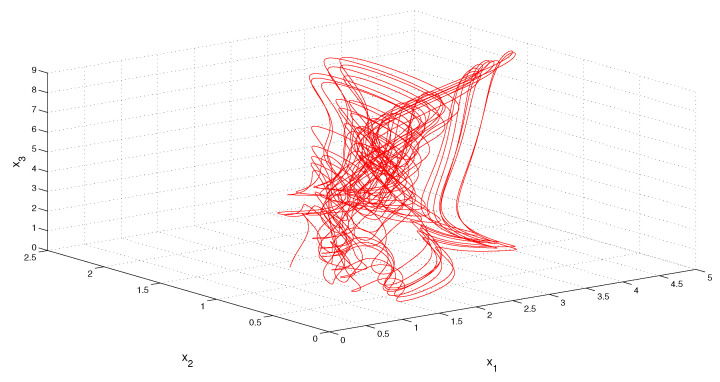
The trajectory of the solution, x(t), of Equation (10), which asymptotically approaches the MPPS solution of the equation.

In the next example, the periodic component ϕ(t) of the MPPS perturbation is absent, but the condition (C2) is correct, since a constant function is of arbitrary period. It is remarkable to say that the absence of a proper non-constant periodic component makes the dynamics more irregular, this is seen in Figure 3 and Figure 4.

**Example** **2.** *Consider the inhomogeneous linear system*

(10) $$\begin{array}{c} \begin{array}{c} x_{1}^{\prime }=\left(-0.25+0.5cos\left(\pi t\right)\right)x_{1}+12\Theta^{3}\left(t\right),\\ x_{2}^{\prime }=\left(-1.5+sin^{2}\left(\pi t\right)\right)x_{2}+8\Theta^{2}\left(t\right),\\ x_{3}^{\prime }=\left(-0.5+cos\left(\frac{2\pi }{3}t\right)\right)x_{3}+6\Theta \left(t\right), \end{array} \end{array}$$

*where $\Theta \left(t\right)=\int_{-\infty }^{t}e^{-2(t-s)}\Omega_{\left(3.9;6\right)}\left(s\right)ds.$ The conditions (C1)–(C3) are satisfied, and condition (C4) is valid with multipliers $\rho_{1}=e^{-0.75},$ $\rho_{2}=e^{-3}$ and $\rho_{3}=e^{-1.5}.$ Consequently, there exists the unique asymptotically stable MPPS solution of the system (10). Figure 3 presents the coordinates of the solution x(t) with initial values $x_{1}\left(0\right)=1,$ $x_{2}\left(0\right)=1$ and $x_{3}\left(0\right)=1.$ The coordinates of solution x(t) approximate the coordinates of the MPPS solution. The trajectory of the solution x(t) is shown in Figure 4.*

### 3.2. Quasilinear Differential Equations

The main object of the present section is the system of quasilinear differential equations

(11) $$\begin{array}{c} x^{\prime }\left(t\right)=A\left(t\right)x+g\left(t,x\right)+\phi \left(t\right)+\psi \left(t\right), \end{array}$$

where $t\in R,x\in R^{n},$*n* is a fixed natural number; A(t) is n− dimensional square matrix and satisfies to the condition (C1) and inequality (3); $g:R\times U\to R^{n},g=\left(g_{1},\ldots ,g_{n}\right),$ $U=\{x\in R^{n},∥x∥<H\},$ where *H* is a fixed positive number; the functions ϕ(t) and ψ(t) satisfy conditions (C2) and (C3).

The following conditions on system (11) are required.

**(C5)** 
the function g(t,x) is continuous and ω− periodic in t;
**(C6)** 
there exists a positive constant *L* such that $∥g\left(t,x_{1}\right)-g\left(t,x_{2}\right)∥\leq L∥x_{1}-x_{2}∥$ for all $t\in R,x_{1},x_{2}\in U.$

We denote $\underset{R\times U}{\sup}∥g\left(t,x\right)∥=m_{g},$ $\max_{t\in R}∥\phi \left(t\right)∥=m_{\phi }$ and $\underset{t\in R}{\sup}∥\psi \left(t\right)∥=m_{\psi }.$

The following additional conditions will be needed:

**(C7)** 
$\frac{K(m_{g}+m_{\phi }+m_{\psi })}{H}<\alpha ;$
**(C8)** 
KL<α.

For simplicity, we use the notation F(t,x)=g(t,x)+ϕ(t)+ψ(t) in what follows.

According to [28], a bounded on the real axis function y(t) is a solution of (11), if and only if it satisfies the equation

(12) $$\begin{array}{ccc} & & y\left(t\right)=\int_{-\infty }^{t}X\left(t,s\right)F\left(s,y\left(s\right)\right)ds,\;t\in R. \end{array}$$

**Theorem** **2.** *If conditions (C1)–(C8) are valid, then the system (11) possesses a unique asymptotically stable Poisson stable solution.*

**Proof.**  Let $t_{k}$ is the Poisson sequence of the function ψ(t) in the system (11). We denote by *B* the set of all Poisson stable functions $\nu \left(t\right)=\left(\nu_{1},\nu_{2},...,\nu_{n}\right),$ $\nu_{i}\in R,$ i=1,2,...,n, with common Poisson sequence $t_{k},$ which satisfy ${∥\nu ∥}_{1}<H.$

Let us show that the *B* is a complete space. Consider a Cauchy sequence $\theta_{m}\left(t\right)$ in *B*, which converges to a limit function θ(t) on R. We have that

(13) $$\begin{array}{ccc} & & ∥\theta \left(t+t_{k}\right)-\theta \left(t\right)∥<∥\theta \left(t+t_{k}\right)-\theta_{m}\left(t+t_{k}\right)∥+∥\theta_{m}\left(t+t_{k}\right)-\theta_{m}\left(t\right)∥+\\ & & ∥\theta_{m}\left(t\right)-\theta \left(t\right)∥. \end{array}$$

for a fixed closed and bounded interval I⊂R. Now, one can take sufficiently large *m* and *k* such that each term on the right hand-side of (13) is smaller than $\frac{\epsilon }{3}$ for a fixed positive ϵ and t∈I, i.e., the sequence $\theta (t+t_{k})$ uniformly converges to θ(t) on I. Likewise, one can check that the limit function is uniformly continuous [28]. The completeness of *B* is shown.

Define the operator Π on *B* such that

(14) $$\begin{array}{ccc} & & \Pi \nu \left(t\right)=\int_{-\infty }^{t}X\left(t,s\right)F\left(s,\nu \left(s\right)\right)ds,\;t\in R. \end{array}$$

Fix a function ν(t) that belongs to *B*. We have that

$$\begin{array}{ccc} & & ∥\Pi \nu \left(t\right)∥\leq \int_{-\infty }^{t}∥X\left(t,s\right)∥∥F\left(s,\nu \left(s\right)\right)∥ds\leq \frac{K(m_{g}+m_{\phi }+m_{\psi })}{\alpha } \end{array}$$

for all t∈R. Therefore, by the condition (C7) it is true that ${∥\Pi \nu ∥}_{1}<H$.

Fix a positive number ϵ and an interval [a,b], −∞<a<b<∞. Let us choose two numbers c<a, and ξ>0 satisfying the inequalities

(15) $$\begin{array}{c} \frac{4K^{2}\xi }{\alpha^{3}e}\left(m_{g}+m_{\phi }+m_{\psi }\right)<\frac{\epsilon }{3}, \end{array}$$

(16) $$\begin{array}{c} \frac{2K}{\alpha }\left(m_{g}+m_{\phi }+m_{\psi }\right)e^{-\alpha (a-c)}<\frac{\epsilon }{3}, \end{array}$$

and

(17) $$\begin{array}{c} \frac{K\xi }{\alpha }\left[1-e^{-\alpha (b-c)}\right]<\frac{\epsilon }{3}. \end{array}$$

Using the condition (C4) and Lemmas A3 and A5 from Appendix A, without loss of generality, we obtain that $∥A\left(t+t_{k}\right)-A\left(t\right)∥<\xi$ for all t∈R, and $∥F\left(t+t_{k},\nu \left(t+t_{k}\right)\right)-F\left(t,\nu \left(t\right)\right)∥<\xi$ for t∈[c,b] and sufficiently large k. Then, applying the inequality (4), we obtain:

$$\begin{array}{ccc} & & ∥\Pi \nu \left(t+t_{k}\right)-\Pi \nu \left(t\right)∥=\\ & & ∥\int_{-\infty }^{t}X\left(t+t_{k},s+t_{k}\right)F\left(s+t_{k},\nu \left(s+t_{k}\right)\right)ds-\int_{-\infty }^{t}X\left(t,s\right)F\left(s,\nu \left(s\right)\right)ds∥\leq \\ & & \int_{-\infty }^{t}∥X\left(t+t_{k},s+t_{k}\right)-X\left(t,s\right)∥∥F\left(s+t_{k},\nu \left(s+t_{k}\right)\right)∥ds+\\ & & \int_{-\infty }^{c}∥X\left(t,s\right)∥∥F\left(s+t_{k},\nu \left(s+t_{k}\right)\right)-F\left(t,s\right)∥ds+\\ & & \int_{c}^{t}∥X\left(t,s\right)∥∥F\left(s+t_{k},\nu \left(s+t_{k}\right)\right)-F\left(t,s\right)∥ds\leq \\ & & \int_{-\infty }^{t}\frac{2K^{2}\xi }{\alpha^{2}e}e^{-\frac{\alpha }{2}\left(t-s\right)}\left(m_{g}+m_{\phi }+m_{\psi }\right)ds+\\ & & \int_{-\infty }^{t}2Ke^{-\alpha (t-s)}\left(m_{g}+m_{\phi }+m_{\psi }\right)ds+\int_{-\infty }^{t}Ke^{-\alpha (t-s)}\xi ds\leq \\ & & \frac{4K\xi }{\alpha^{3}e}\left(m_{g}+m_{\phi }+m_{\psi }\right)+\frac{2K}{\alpha }\left(m_{g}+m_{\phi }+m_{\psi }\right)e^{-\alpha (a-c)}+\frac{K\xi }{\alpha }\left[1-e^{-\alpha (b-c)}\right], \end{array}$$

for all t∈[a,b]. From inequalities (15)–(17) it follows that $∥\Pi \nu \left(t+t_{k}\right)-\Pi \nu \left(t\right)∥<\epsilon$ for t∈[a,b]. Therefore, $\Pi \nu (t+t_{k})$ uniformly converges to Πν(t) on bounded interval of R.

It is easy to verify that Πν(t) is a uniformly continuous function, since its derivative is a uniformly bounded function on the real axis. Summarizing the above discussion, the set *B* is invariant for the operator Π.

We proceed to show that the operator Π:B→B is contractive. Let u(t) and v(t) be members of *B*. Then, we obtain that

$$\begin{array}{ccc} & & ∥\Pi u\left(t\right)-\Pi v\left(t\right)∥\leq \int_{-\infty }^{t}∥X\left(t,s\right)∥∥F\left(s,u\left(s\right)\right)-F\left(s,v\left(s\right)\right)∥ds\leq \\ & & \int_{-\infty }^{t}Ke^{-\alpha (t-s)}L∥u\left(s\right)-v\left(s\right)∥ds\leq \frac{KL}{\alpha }{∥u\left(t\right)-v\left(t\right)∥}_{1}, \end{array}$$

for all t∈R. Therefore, the inequality ${∥\Pi u-\Pi v∥}_{1}\leq \frac{KL}{\alpha }{∥u-v∥}_{1}$ holds, and according to the condition (C8) the operator Π:B→B is contractive.

By the contraction mapping theorem there exists the unique fixed point, $\bar{x}\left(t\right)\in B,$ of the operator Π, which is the unique bounded Poisson stable solution of the system (11).

Finally, we will study the asymptotic stability of the Poisson stable solution $\bar{x}\left(t\right)$ of the system (11). It is true that

$$\begin{array}{c} \bar{x}\left(t\right)=X\left(t,t_{0}\right)\bar{x}\left(t_{0}\right)+\int_{t_{0}}^{t}X\left(t,s\right)\left(g(s,\bar{x}\left(s\right))+\phi \left(s\right)+\psi \left(s\right)\right)ds, \end{array}$$

for $t\geq t_{0}.$

Let x(t) be another solution of system (11). One can write

$$\begin{array}{c} x\left(t\right)=X\left(t,t_{0}\right)x\left(t_{0}\right)+\int_{t_{0}}^{t}X\left(t,s\right)\left(g(s,x(s))+\phi (s)+\psi (s)\right)ds. \end{array}$$

Making use of the relation

$$\begin{array}{c} \bar{x}\left(t\right)-x\left(t\right)=X\left(t,t_{0}\right)\left(\bar{x}\left(t_{0}\right)-x\left(t_{0}\right)\right)+\int_{t_{0}}^{t}X\left(t,s\right)\left(g\left(s,\bar{x}\left(s\right)\right)-g\left(s,x\left(s\right)\right)\right)ds, \end{array}$$

we obtain that

$$\begin{array}{ccc} & & ∥\bar{x}\left(t\right)-x\left(t\right)∥\leq ∥X\left(t,t_{0}\right)∥∥\bar{x}\left(t_{0}\right)-x\left(t_{0}\right)∥+\int_{t_{0}}^{t}∥X\left(t,s\right)∥∥g\left(s,\bar{x}\left(s\right)\right)-g(s,x\left(s\right)∥ds\leq \\ & & Ke^{-\alpha (t-t_{0})}∥\bar{x}\left(t_{0}\right)-x\left(t_{0}\right)∥+\int_{t_{0}}^{t}KLe^{-\alpha (t-s)}∥\bar{x}\left(s\right)-x\left(s\right)∥ds. \end{array}$$

Now, applying Gronwall–Bellman Lemma, one can attain that

(18) $$\begin{array}{ccc} & & ∥\bar{x}\left(t\right)-x\left(t\right)∥\leq Ke^{-\left(\alpha -KL\right)\left(t-t_{0}\right)}∥\bar{x}\left(t_{0}\right)-x\left(t_{0}\right)∥,t\geq t_{0}. \end{array}$$

The last inequality and condition (C8) confirm that the Poisson stable solution $\bar{x}\left(t\right)$ is asymptotically stable. The theorem is proved.  □

**Remark** **2.** *According to the Lemma A4 in the Appendix A, the Poisson stable solution $\bar{x}\left(t\right)$ of the system (11) is an MPPS function.*

**Example** **3.** *Consider the quasilinear system.*

(19) $$\begin{array}{ccc} & & \begin{array}{c} x_{1}^{\prime }=\left(-1.5+2sin\left(2t\right)\right)x_{1}+0.01cos\left(2t\right)arctg\left(x_{2}\right)+1.2sin\left(8t\right)-10.5\Theta^{3}\left(t\right),\\ x_{2}^{\prime }=\left(-3.5+3sin^{2}\left(2t\right)\right)x_{2}+0.03sin\left(4t\right)arctg\left(x_{3}\right)-1.5cos\left(8t\right)+2.5\Theta \left(t\right),\\ x_{3}^{\prime }=\left(-1.5+2cos^{2}\left(t\right)\right)x_{3}-0.02sin\left(2t\right)arctg\left(x_{1}\right)+sin\left(4t\right)+7.2\Theta^{2}\left(t\right), \end{array} \end{array}$$

*where $\Theta \left(t\right)=\int_{-\infty }^{t}e^{-3(t-s)}\Omega_{\left(3.86,3\pi \right)}\left(s\right)ds$ is the Poisson stable function, which described similarly to that in Appendix B. Since, the piecewise constant function $\Omega_{\left(3.86;3\pi \right)}\left(t\right)$ is given on intervals [3πi,3π(i+1)), for the Poisson sequence $t_{k}$ of the function Θ(t) there exists a subsequence $t_{k_{l}}$ such that $t_{k_{l}}\to 0\left(mod\;\pi \right),$ that is the condition (C3) is valid. The common period of the matrix A(t) and functions g(t,x), ϕ(t) is equal to π. We have that the function $g\left(t,x\right)={\left(0.01cos\left(2t\right)arctg\left(x_{2}\right),0.03sin\left(4t\right)arctg\left(x_{3}\right),-0.02sin\left(2t\right)arctg\left(x_{1}\right)\right)}^{T}$ is continuous and π− periodic in t and satisfies condition (C6) with L=0.03. The sum of $\phi \left(t\right)={\left(1.2sin\left(8t\right),-1.5cos\left(8t\right),sin\left(4t\right)\right)}^{T}$ and $\psi \left(t\right)={\left(10.5\Theta^{3}\left(t\right),2.5\Theta \left(t\right),7.2\Theta^{2}\left(t\right)\right)}^{T}$ is an MPPS function, which meets conditions (C2), (C3). The assumptions (C4)–(C8) are valid with $m_{g}=0.048,$ $m_{\phi }=1.5,$ $m_{\psi }=0.84,$ $\rho_{1}=e^{-1.5\pi },$ $\rho_{2}=e^{-2\pi },$ $\rho_{3}=e^{-0.5\pi },$ α=0.5π, K=1, and H=4.8. Thus, all conditions for the last theorem have been verified, and there is the Poisson stable solution of the system, which is asymptotically stable.*

**Figure 5:**
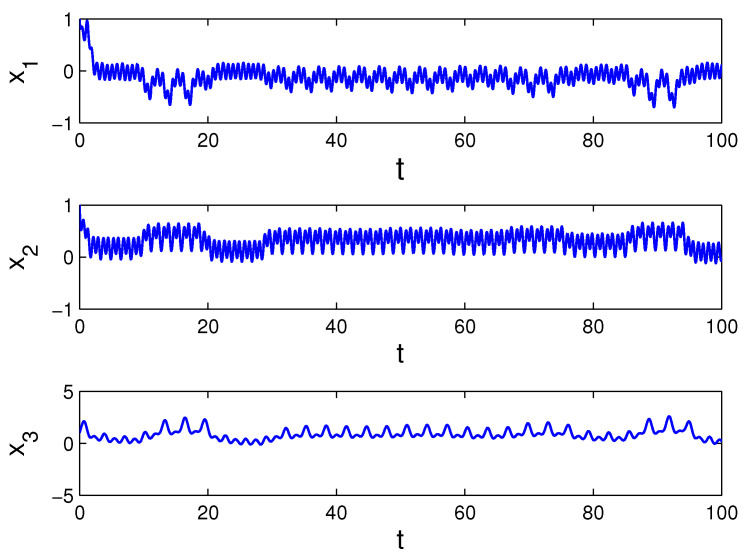
The coordinates of the solution x(t), with $x_{1}\left(0\right)=1,$ $x_{2}\left(0\right)=1,$ $x_{3}\left(0\right)=1,$ which is asymptotic for the Poisson stable solution of the system (19).

**Figure 6:**
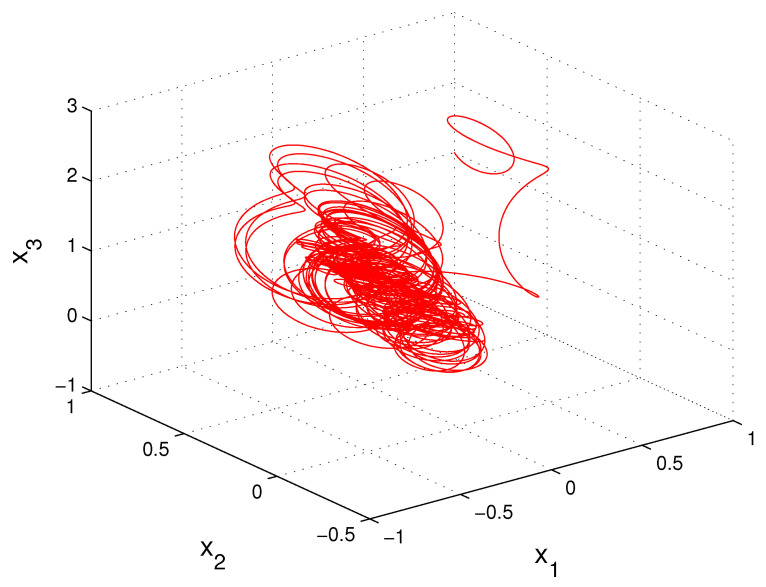
The trajectory of the solution x(t), with $x_{1}\left(0\right)=1,$ $x_{2}\left(0\right)=1,$ $x_{3}\left(0\right)=1,$ which illustrates the Poisson stability of the system (19).

It is worth noting that the simulation of the Poisson stable solution, $\bar{x}\left(t\right),$ is not possible, since the initial value is not known precisely. For this reason, we will consider the solution x(t) of the system (19), with initial values $x_{1}\left(0\right)=1,$ $x_{2}\left(0\right)=1$ and $x_{3}\left(0\right)=1.$ Using the inequality (18) one can obtain that $∥\bar{x}\left(t\right)-x\left(t\right)∥\leq e^{-1.54}∥\bar{x}\left(0\right)-x\left(0\right)∥$ for t≥0. The last inequality shows that $∥\bar{x}\left(t\right)-x\left(t\right)∥$ decreases exponentially. Consequently, the graph of the solution x(t) asymptotically approaches the Poisson stable solution $\bar{x}\left(t\right)$ of the system (19), as time increases. The Figure 5 demonstrates the coordinates of the solution x(t), which illustrate the Poisson stability of the system (19). In the Figure 6 the trajectory of the function x(t) is depicted.

### 3.3. A Case with MPPS Coefficients

Let us consider the quasilinear Equation (11) with A(t)=B(t)+D(t), where B(t) is a continuous ω− periodic matrix, and D(t) is a Poisson stable matrix with the Poisson sequence $t_{k}.$ That is, the coefficient is an MPPS matrix and the system (11) is of the form

(20) $$\begin{array}{c} x^{\prime }\left(t\right)=\left(B\left(t\right)+D\left(t\right)\right)x+g\left(t,x\right)+\phi \left(t\right)+\psi \left(t\right), \end{array}$$

where the functions ϕ(t) and ψ(t) satisfy conditions (C2) and (C3) and their sum is an MPPS function. The function g(t,x) satisfies conditions (C5), (C6).

Denote G(t,x)=D(t)x+g(t,x)+ϕ(t)+ψ(t) and rewrite the system (20) as

(21) $$\begin{array}{c} x^{\prime }\left(t\right)=B\left(t\right)x+G\left(t,x\right). \end{array}$$

The homogeneous ω− periodic system, associated with (20),

(22) $$\begin{array}{c} y^{\prime }\left(t\right)=B\left(t\right)y, \end{array}$$

has the fundamental matrix Y(t), Y(0)=I, and the transition matrix Y(t,s), t,s∈R.

Assume that the following assumptions are valid.

**(C9)**  The multipliers of the system (22) are in modulus less than one.

From the condition (C9) we have that there exist positive numbers D≥1 and β such that

(23) $$\begin{array}{c} ∥Y\left(t,s\right)∥\leq De^{-\beta (t-s)}, \end{array}$$

for t≥s.

**(C10)** 
D(L+d)<β;
**(C11)** 
$\frac{D(m_{g}+m_{\phi }+m_{\psi })}{H}<\beta -Dd,$

where $d=\sup_{t\in R}∥D\left(t\right)∥.$

**Theorem** **3.** *If conditions (C2), (C3), (C5), (C6), and (C9) to (C11) are hold, then system (20) admits a unique asymptotically stable Poisson stable solution.*

**Proof.**  A bounded on the real axis function z(t) is a solution of (21), if and only if it satisfies the equation

(24) $$\begin{array}{ccc} & & z\left(t\right)=\int_{-\infty }^{t}Y\left(t,s\right)G\left(s,z\left(s\right)\right)ds,\;t\in R. \end{array}$$

Denote by U the Banach space of all Poisson stable functions $\nu \left(t\right)=\left(\nu_{1},\nu_{2},...,\nu_{n}\right),$ $\nu_{i}\in R,$ i=1,2,...,n, with common Poisson sequence $t_{k}.$ The functions of space U satisfies the condition ${∥\nu ∥}_{1}<H.$

Introduce the operator Γ on U such that

(25) $$\begin{array}{ccc} & & \Gamma \nu \left(t\right)=\int_{-\infty }^{t}Y\left(t,s\right)G\left(s,\nu \left(s\right)\right)ds,\;t\in R. \end{array}$$

Let us show that the space U is invariant for the operator Γ. Fix a function ν(t) from U. We have that

$$\begin{array}{ccc} & & ∥\Gamma \nu \left(t\right)∥\leq \int_{-\infty }^{t}∥Y\left(t,s\right)∥∥G\left(s,\nu \left(s\right)\right)∥ds\leq \frac{D(dH+m_{g}+m_{\phi }+m_{\psi })}{\beta } \end{array}$$

for all t∈R. Condition (C11) implies that ${∥\Gamma \nu ∥}_{1}<H$.

Next, we will use fixed positive number ϵ and an interval [a,b], −∞<a<b<∞, and two numbers c<a, and ξ>0 satisfying the following inequalities

(26) $$\begin{array}{c} \frac{4DK^{2}\xi }{\beta^{3}e}\left(dH+m_{g}+m_{\phi }+m_{\psi }\right)<\frac{\epsilon }{3}, \end{array}$$

(27) $$\begin{array}{c} \frac{2D}{\beta }\left(dH+m_{g}+m_{\phi }+m_{\psi }\right)e^{-\alpha (a-c)}<\frac{\epsilon }{3}, \end{array}$$

and

(28) $$\begin{array}{c} \frac{D\xi }{\beta }\left[1-e^{-\alpha (b-c)}\right]<\frac{\epsilon }{3}. \end{array}$$

Using the condition (C9) and Lemmas A3, A5 from Appendix A, we obtain that $∥B\left(t+t_{k}\right)-B\left(t\right)∥<\xi$ for all t∈R, and $∥G\left(t+t_{k},\nu \left(t+t_{k}\right)\right)-G\left(t,\nu \left(t\right)\right)∥<\xi$ for t∈[c,b] and sufficiently large k. Then, applying the inequality (4), we obtain

$$\begin{array}{ccc} & & ∥\Gamma \nu \left(t+t_{k}\right)-\Gamma \nu \left(t\right)∥=\\ & & ∥\int_{-\infty }^{t}Y\left(t+t_{k},s+t_{k}\right)G\left(s+t_{k},\nu \left(s+t_{k}\right)\right)ds-\int_{-\infty }^{t}Y\left(t,s\right)G\left(s,\nu \left(s\right)\right)ds∥\leq \\ & & \int_{-\infty }^{t}∥Y\left(t+t_{k},s+t_{k}\right)-Y\left(t,s\right)∥∥G\left(s+t_{k},\nu \left(s+t_{k}\right)\right)∥ds+\\ & & \int_{-\infty }^{c}∥Y\left(t,s\right)∥∥G\left(s+t_{k},\nu \left(s+t_{k}\right)\right)-G\left(t,s\right)∥ds+\\ & & \int_{c}^{t}∥Y\left(t,s\right)∥∥G\left(s+t_{k},\nu \left(s+t_{k}\right)\right)-G\left(t,s\right)∥ds\leq \\ & & \int_{-\infty }^{t}\frac{2D^{2}\xi }{\beta^{2}e}e^{-\frac{\beta }{2}\left(t-s\right)}\left(dH+m_{g}+m_{\phi }+m_{\psi }\right)ds+\\ & & \int_{-\infty }^{t}2De^{-\beta (t-s)}\left(dH+m_{g}+m_{\phi }+m_{\psi }\right)ds+\int_{-\infty }^{t}De^{-\beta (t-s)}\xi ds\leq \\ & & \frac{4D\xi }{\beta^{3}e}\left(dH+m_{g}+m_{\phi }+m_{\psi }\right)+\frac{2D}{\beta }\left(dH+m_{g}+m_{\phi }+m_{\psi }\right)e^{-\beta (a-c)}+\frac{D\xi }{\beta }\left[1-e^{-\beta (b-c)}\right], \end{array}$$

for all t∈[a,b]. Hence, the inequalities (26)–(28) give that $∥\Gamma \nu \left(t+t_{k}\right)-\Gamma \nu \left(t\right)∥<\epsilon$ for t∈[a,b]. Therefore, the sequence $\Gamma \nu (t+t_{k})$ uniformly converges to Γν(t) on the bounded interval of R. Thus, we have shown that the operator Γ is invariant in U.

Let us show that the operator Γ:U→U is contractive. Fix members u(t) and v(t) of U. It is true that

$$\begin{array}{ccc} & & ∥\Gamma u\left(t\right)-\Gamma v\left(t\right)∥\leq \int_{-\infty }^{t}∥Y\left(t,s\right)∥∥G\left(s,u\left(s\right)\right)-G\left(s,v\left(s\right)\right)∥ds\leq \\ & & \int_{-\infty }^{t}De^{-\beta (t-s)}\left(d+L\right)∥u\left(s\right)-v\left(s\right)∥ds\leq \frac{D(d+L)}{\beta }{∥u\left(t\right)-v\left(t\right)∥}_{1}, \end{array}$$

for all t∈R, and condition (C10) implies that the operator Γ is contractive.

Using the contraction mapping theorem, one can conclude that there exists a unique fixed point, $\bar{x}\left(t\right),$ of the operator Γ, which is the Poisson stable solution of the system (20). Let us investigate its stability.

If x(t) is a solution of the Equation (20), then

$$\begin{array}{ccc} & & \bar{x}\left(t\right)-x\left(t\right)=Y\left(t,t_{0}\right)\left(\bar{x}\left(t_{0}\right)-x\left(t_{0}\right)\right)+\\ & & \int_{t_{0}}^{t}Y\left(t,s\right)\left(D\left(s\right)\left(\bar{x}\left(s\right)-x\left(s\right)\right)+(g\left(s,\bar{x}\left(s\right)\right)-g\left(s,x\left(s\right)\right)\right)ds, \end{array}$$

and

$$\begin{array}{ccc} & & ∥\bar{x}\left(t\right)-x\left(t\right)∥\leq ∥Y\left(t,t_{0}\right)∥∥\bar{x}\left(t_{0}\right)-x\left(t_{0}\right)∥+\\ & & \int_{t_{0}}^{t}∥Y\left(t,s\right)∥\left(∥D\left(s\right)\left(\bar{x}\left(s\right)-x\left(s\right)\right)∥+∥g\left(s,\bar{x}\left(s\right)\right)-g(s,x\left(s\right)∥\right)ds\leq \\ & & De^{-\beta (t-t_{0})}∥\bar{x}\left(t_{0}\right)-x\left(t_{0}\right)∥+\int_{t_{0}}^{t}D\left(d+L\right)e^{-\alpha (t-s)}∥\bar{x}\left(s\right)-x\left(s\right)∥ds. \end{array}$$

With the aid of the Gronwall–Bellman Lemma, one can verify that

(29) $$\begin{array}{ccc} & & ∥\bar{x}\left(t\right)-x\left(t\right)∥\leq De^{-\left(\beta -D\left(d+L\right)\right)\left(t-t_{0}\right)}∥\bar{x}\left(t_{0}\right)-x\left(t_{0}\right)∥,t\geq t_{0}. \end{array}$$

Now, based on the condition (C10), we conclude that the Poisson stable solution $\bar{x}\left(t\right)$ of system (20) is asymptotically stable. The theorem is proved.  □

## 4. Conclusions

In this paper, we have introduced a new type of recurrence, which is the sum of two compartments, periodic and Poisson stable functions. We call it as modulo periodic Poisson stable function. Sufficient conditions for the dynamics to be Poisson stable have been determined. The novelty is convenient for theoretical analysis of differential and discrete equations of various types. In the present paper, we study quasilinear ordinary differential equations. If one consider the periodic compartment in the Poisson stability, and achievements of the paper for simulations of the recurrence, the results create new productive opportunities in the research of mechanical, electronic dynamics and neuroscience. Concerning theoretical research, it is of strong interest to search for Poisson stability and its periodic components in such famous dynamics as Lorenz, Rössler and Chua attractors. Generally speaking, one can look for periodic components of any chaotic dynamics. The results can be applied in problems of optimization. The results can be applied for problems of optimization. 

## Data Availability

Data is contained within the article.

## Appendix A

**Lemma** **A1.**
*For arbitrary sequence of positive real numbers $t_{k},$ k=1,2,⋯, and a positive number ω there exist a subsequence $t_{k_{l}},$ l=1,2,⋯, and a number $\tau_{\omega },$ $0\leq \tau_{\omega }<\omega ,$ such that $t_{k_{l}}\to \tau_{\omega }\left(mod\;\omega \right)$ as l→∞.*

**Proof.** Consider the sequence $\tau_{k}$ such that $t_{k}\equiv \tau_{k}\left(mod\;\omega \right),$ and $0\leq \tau_{k}<\omega$ for all k≥1. The boundedness of the sequence $\tau_{k}$ implies that there exists a subsequence $\tau_{k_{l}},$ which converges to a number $\tau_{\omega }$ [30].  □

**Lemma** **A2.**
*
$\kappa_{\omega }\in T_{\omega }.$
*

**Proof.** Assume on the contrary that $\kappa_{\omega }$ is not in $T_{\omega }.$ Then there exists a strictly decreasing sequence $\tau_{m},$ m≥1, in $T_{\omega },$ such that $\tau_{m}\to \kappa_{\omega }.$ For each natural m, denote by $t_{i}^{m}$ a subsequence of $t_{k}$ such that $t_{i}^{m}\to \tau_{m}\left(mod\;\omega \right)$ as i→∞. Fix a sequence of positive numbers $\epsilon_{n},$ which converges to the zero. One can find numbers $i_{n},$ n=1,2,…, such that $|t_{i_{n}}^{n}-\tau_{n}|<\epsilon_{n}\left(mod\;\omega \right).$ It is clear that $t_{i_{n}}^{n}\to \kappa_{\omega }\left(mod\;\omega \right)$ as n→∞.  □

**Remark** **A1.**
*The last assertion implies that if $\kappa_{\omega }=0,$ then there exists a subsequence $t_{k_{l}}$ such that $t_{k_{l}}\to 0\left(mod\;\omega \right)$ as l→∞.*

**Lemma** **A3.**
*If f(t)=ϕ(t)+ψ(t) is an MPPS function, and $\kappa_{\omega }=0,$ then the function f(t) is Poisson stable.*

**Proof.** According to Lemma A2, there exists a subsequence $t_{k_{l},}$ which tends to zero in modulus ω as l→∞. Without loss of generality assume that $t_{k}\to 0\left(mod\;\omega \right)$ as k→∞. Fix a positive number ϵ, and bounded interval I⊂R. The periodic function ϕ(t) is uniformly continuous on R. Consequently, there exists a number $k_{1}$ such that

$$\begin{array}{c} ∥\phi \left(t+t_{k}\right)-\phi \left(t\right)∥<\frac{\epsilon }{2}, \end{array}$$

for all t∈R and $k>k_{1}.$ Moreover, there exists an integer $k_{2},$ such that

$$\begin{array}{c} ∥\psi \left(t+t_{k}\right)-\psi \left(t\right)∥<\frac{\epsilon }{2}, \end{array}$$

for t∈I, $k>k_{2}.$ This is why,

$$\begin{array}{ccc} & & ∥f\left(t+t_{k}\right)-f\left(t\right)∥\leq ∥\phi \left(t+t_{k}\right)-\phi \left(t\right)∥+∥\psi \left(t+t_{k}\right)-\psi \left(t\right)∥<\epsilon , \end{array}$$

if t∈I and $k>\max(k_{1},k_{2}).$ That is, the function f(t) is Poisson stable.  □

**Lemma** **A4.**
*Assume that ψ(t) is a Poisson stable function. If $\kappa_{\omega }=0,$ for some positive number ω, then ψ(t) is MPPS function.*

**Proof.** Let us write ψ(t)=g(t)+(ψ(t)−g(t)), where g(t) is a continuous ω− periodic function. Since $\kappa_{\omega }=0,$ then the subtraction ψ(t)−g(t) is Poisson stable by Lemma A3.  □

**Remark** **A2.**
*The last result is a source for the optimization problem how to choose the function g(t) and the period ω to minimize the difference ψ(t)−g(t). In other words, the problem of approximation of Poisson stable functions with periodic ones. It is of exceptional interest for celestial mechanics [2].*

**Lemma** **A5.**
*Assume that a function $G\left(t,u\right):R\times U\to R^{n},U\subseteq R^{n},$ is a Poisson stable function in t and satisfies the inequality $∥G\left(t,u_{1}\right)-G\left(t,u_{2}\right)∥\leq L∥u_{1}-u_{2}∥,$ where L is a positive constant, for all $t\in R,u_{1},u_{2}\in U.$ Moreover, υ(t):R→U is ω− periodic in t. If the Poisson sequence and period ω are such that the Poisson number $\kappa_{\omega }$ equals to the zero, then the function G(t,υ(t)) is Poisson stable.*

**Proof.** By the Lemma A2 there exists a subsequence $t_{k_{l}},$ such that $t_{k_{l}}\to 0\left(mod\;\omega \right)$ as l→∞. We assume, without loss of generality, that the sequence $t_{k}$ itself satisfies the condition $t_{k}\to 0\left(mod\;\omega \right)$ as k→∞. Let us fix a positive number ϵ, and a bounded interval I. Since of the property of the sequence $t_{k},$ we have that for sufficiently large k, it is true that $∥G\left(t+t_{k},\upsilon \left(t+t_{k}\right)\right)-G\left(t,\upsilon \left(t+t_{k}\right)\right)∥<\frac{\epsilon }{2}$ for all t∈R, and $∥\upsilon \left(t+t_{k}\right)-\upsilon \left(t\right)∥<\frac{\epsilon }{2L}$ for t∈I, and

$$\begin{array}{ccc} & & ∥G\left(t+t_{k},\upsilon \left(t+t_{k}\right)\right)-G\left(t,\upsilon \left(t\right)\right)∥\leq ∥G\left(t+t_{k},\upsilon \left(t+t_{k}\right)\right)-G\left(t,\upsilon \left(t+t_{k}\right)\right)∥+\\ & & ∥G\left(t,\upsilon \left(t+t_{k}\right)\right)-G\left(t,\upsilon \left(t\right)\right)∥\leq ∥G\left(t+t_{k},\upsilon \left(t+t_{k}\right)\right)-G\left(t,\upsilon \left(t+t_{k}\right)\right)∥+\\ & & L∥\upsilon \left(t+t_{k}\right)-\upsilon \left(t\right)∥\leq \frac{\epsilon }{2}+L\frac{\epsilon }{2L}\leq \epsilon , \end{array}$$

for all t∈I. That is, G(t,υ(t)) is Poisson stable function.  □

**Lemma** **A6.**
*Assume that a function $G\left(t,u\right):R\times U\to R^{n},U\subseteq R^{n},$ is ω− periodic in t and satisfies the inequality $∥G\left(t,u_{1}\right)-G\left(t,u_{2}\right)∥\leq L∥u_{1}-u_{2}∥,$ where L is a positive constant, for all $t\in R,u_{1},u_{2}\in U.$ Moreover, υ(t):R→U is a Poisson stable function. If the Poisson sequence and period ω are such that the Poisson number $\kappa_{\omega }$ equals to the zero, then the function G(t,υ(t)) is Poisson stable.*

**Proof.** Since $\kappa_{\omega }=0,$ the Lemma A2 implies that there exists a subsequence $t_{k_{l}},$ such that $t_{k_{l}}\to 0\left(mod\;\omega \right)$ as l→∞. For simplicity, we assume that the sequence $t_{k}$ itself satisfies the condition $t_{k}\to 0\left(mod\;\omega \right)$ as k→∞. Therefore, $G(t+t_{k},v)$ uniformly converges to G(t,v) as k→∞, for all t∈R and v∈U. Consequently, for arbitrarily fixed positive number ϵ and a bounded interval *I* one can find sufficiently large number *k* such that $∥G\left(t+t_{k},\upsilon \left(t+t_{k}\right)\right)-G\left(t,\upsilon \left(t+t_{k}\right)\right)∥<\frac{\epsilon }{2}$ for all t∈R, and $∥\upsilon \left(t+t_{k}\right)-\upsilon \left(t\right)∥<\frac{\epsilon }{2L}$ for t∈I. Finally, we have that

$$\begin{array}{ccc} & & ∥G\left(t+t_{k},\upsilon \left(t+t_{k}\right)\right)-G\left(t,\upsilon \left(t\right)\right)∥\leq ∥G\left(t+t_{k},\upsilon \left(t+t_{k}\right)\right)-G\left(t,\upsilon \left(t+t_{k}\right)\right)∥+\\ & & ∥G\left(t,\upsilon \left(t+t_{k}\right)\right)-G\left(t,\upsilon \left(t\right)\right)∥\leq ∥G\left(t+t_{k},\upsilon \left(t+t_{k}\right)\right)-G\left(t,\upsilon \left(t+t_{k}\right)\right)∥+\\ & & L∥\upsilon \left(t+t_{k}\right)-\upsilon \left(t\right)∥\leq \frac{\epsilon }{2}+L\frac{\epsilon }{2L}\leq \epsilon , \end{array}$$

for all t∈I. That is, G(t,υ(t)) is Poisson stable function.  □

**Lemma** **A7.**
*Assume that a function $G\left(t,u\right):R\times U\to R^{n},U\subseteq R^{n},$ is Poisson stable in t and satisfies the inequality $∥G\left(t,u_{1}\right)-G\left(t,u_{2}\right)∥\leq L∥u_{1}-u_{2}∥,$ where L is a positive constant, for all $t\in R,u_{1},u_{2}\in U.$ Moreover, υ(t):R→U is a Poisson stable function. If there exists a Poisson sequence common for the functions G(t,u) and υ(t), then the function G(t,υ(t)) is Poisson stable.*

**Proof.** Let us fix a positive number ϵ, and a bounded interval I. Since G(t,v(t)) is Poisson stable in t, and v(t) is Poisson stable function, there exists sufficiently large k, such that $∥G\left(t+t_{k},\upsilon \left(t+t_{k}\right)\right)-G\left(t,\upsilon \left(t+t_{k}\right)\right)∥<\frac{\epsilon }{2}$ for all t∈R, and $∥\upsilon \left(t+t_{k}\right)-\upsilon \left(t\right)∥<\frac{\epsilon }{2L}$ for t∈I. That is,

$$\begin{array}{ccc} & & ∥G\left(t+t_{k},\upsilon \left(t+t_{k}\right)\right)-G\left(t,\upsilon \left(t\right)\right)∥\leq ∥G\left(t+t_{k},\upsilon \left(t+t_{k}\right)\right)-G\left(t,\upsilon \left(t+t_{k}\right)\right)∥+\\ & & ∥G\left(t,\upsilon \left(t+t_{k}\right)\right)-G\left(t,\upsilon \left(t\right)\right)∥\leq ∥G\left(t+t_{k},\upsilon \left(t+t_{k}\right)\right)-G\left(t,\upsilon \left(t+t_{k}\right)\right)∥+\\ & & L∥\upsilon \left(t+t_{k}\right)-\upsilon \left(t\right)∥\leq \frac{\epsilon }{2}+L\frac{\epsilon }{2L}\leq \epsilon , \end{array}$$

for all t∈I. Thus, G(t,υ(t)) is Poisson stable function.  □

**Remark** **A3.**
*The last lemma implies, in particular, that sum and product of Poisson stable functions with common Poisson sequence are Poisson stable functions.*

## Appendix B

This part of the paper is about an example of the Poisson stable functions. The task is not an easy one, and there are very few constructively determined cases [4,5]. In our research, we use the dynamical approach of functions determination. One of the most familiar is of sin and cos functions as solutions of ordinary differential equations. We shall consider the Poisson function as a continuous component of solution for a hybrid system, which consists of a discrete equation and a simple differential equation, while discrete component can be accepted as a Poisson stable sequence. A significant element of the present study is visualization of the continuous Poisson stable solution through a neighboring it by an asymptotically close counterpart.

In [6] as a part of the result construction of a Poisson stable sequence was performed as the solution of the logistic equation

(A1) $$\begin{array}{ccc} & & \lambda_{n+1}=\mu \lambda_{n}\left(1-\lambda_{n}\right). \end{array}$$

More precisely, it is proved that for each $\mu \in [3+{\left(2/3\right)}^{1/2},4]$ there exists a solution $\{\eta_{n}\},$ n∈Z, of Equation (A1) such that the sequence belongs to the interval [0,1] and there exists a sequence $\zeta_{n},$ which diverges to infinity such that $|\eta_{i+\zeta_{n}}-\eta_{i}|\to 0$ as n→∞ for each *i* in bounded intervals of integers.

Consider the following integral

(A2) $$\begin{array}{c} \Theta \left(t\right)=\int_{-\infty }^{t}e^{-2(t-s)}\Omega \left(s\right)ds,\;t\in R, \end{array}$$

where Ω(t) is a piecewise constant function defined on the real axis through the equation $\Omega \left(t\right)=\eta_{i}$ for t∈[i,i+1), i∈Z. It is convenient to consider the function Θ(t) as a unique bounded on the real axis solution of the equation $\Theta^{\prime }=-2\Theta +\Omega \left(t\right).$ In all next examples of the paper we use the function notation $\Omega \left(t\right)=\Omega_{\left(\mu ,q\right)}\left(t\right),$ where *q* denotes the length of the intervals on which the function Ω(t) is built.

It is worth noting that Θ(t) is bounded on the hole real axis such that $\sup_{t\in R}\left|\Theta \left(t\right)\right|\leq \;1/2.$

Next, we will show that Θ(t) is a Poisson stable function.

Consider a fixed closed interval [a,b] of the axis and a positive number ε. Without loss of generality one can assume that *a* and *b* are integers. Let us fix a positive number ξ and an integer c<a, which satisfy the following inequalities $e^{-2(a-c)}<\frac{\varepsilon }{2}$ and $\xi [1-e^{-2(b-c)}]<\varepsilon .$ Let *n* be a large natural number such that $|\Omega_{\left(3.89,1\right)}\left(t+\zeta_{n}\right)-\Omega_{\left(3.89,1\right)}\left(t\right)|<\xi$ on [c,b]. Then for all t∈[a,b] we obtain that

$$\begin{array}{ccc} & & |\Theta \left(t+\zeta_{n}\right)-\Theta \left(t\right)\left|=\right|\int_{-\infty }^{t}e^{-2(t-s)}\left(\Omega_{\left(3.89,1\right)}\left(s+\zeta_{n}\right)-\Omega_{\left(3.89,1\right)}\left(s\right)\right)ds|=\\ & & |\int_{-\infty }^{c}e^{-2(t-s)}\left(\Omega_{\left(3.89,1\right)}\left(s+\zeta_{n}\right)-\Omega_{\left(3.89,1\right)}\left(s\right)\right)ds+\\ & & \int_{c}^{t}e^{-2(t-s)}\left(\Omega_{\left(3.89,1\right)}\left(s+\zeta_{n}\right)-\Omega_{\left(3.89,1\right)}\left(s\right)\right)ds|\leq \\ & & \int_{-\infty }^{c}e^{-2(t-s)}2ds+\int_{c}^{b}e^{-2(t-s)}\xi ds\leq e^{-2(a-c)}+\frac{\xi }{2}\left[1-e^{-2(b-c)}\right]<\frac{\varepsilon }{2}+\frac{\varepsilon }{2}=\varepsilon . \end{array}$$

Thus, $|\Theta \left(t+\zeta_{n}\right)-\Theta \left(t\right)|\to 0$ as n→∞ uniformly on the interval [a,b].
